# SARS-CoV-2-Induced Gut Microbiome Dysbiosis: Implications for Colorectal Cancer

**DOI:** 10.3390/cancers13112676

**Published:** 2021-05-28

**Authors:** Mark C. Howell, Ryan Green, Andrew R. McGill, Rinku Dutta, Subhra Mohapatra, Shyam S. Mohapatra

**Affiliations:** 1 Department of Veterans Affairs, James A. Haley Veterans Hospital, Tampa, FL 33612, USA; mhowell1@usf.edu (M.C.H.); rjgreen@usf.edu (R.G.); armcgill@usf.edu (A.R.M.); 2 Department of Internal Medicine, Morsani College of Medicine, University of South Florida, Tampa, FL 33612, USA; 3 Department of Molecular Medicine, Morsani College of Medicine, University of South Florida, Tampa, FL 33612, USA; rinku@usf.edu

**Keywords:** colorectal cancer, COVID-19, gut microbiome, SARS-CoV-2, butyrate

## Abstract

**Simple Summary:**

Specific guidance regarding cancer treatment in coronavirus disease-19 (COVID-19) patients is lacking due to minimal knowledge. It has been observed that patients with severe COVID-19 develop a dysbiotic microbiota of the gut. This impact may be long-lasting, resulting in a greater possibility of a future diagnosis of colorectal cancer (CRC) or aggravating the condition in those already afflicted. Given that CRC is the third most common and third deadliest type of cancer, we must understand how infection with SARS-CoV-2 will impact CRC biology and treatment strategies.

**Abstract:**

The emergence of a novel coronavirus, severe acute respiratory syndrome coronavirus-2 (SARS-CoV-2), in December 2019 led to a worldwide pandemic with over 170 million confirmed infections and over 3.5 million deaths (as of May 2021). Early studies have shown higher mortality rates from SARS-CoV-2 infection in cancer patients than individuals without cancer. Herein, we review the evidence that the gut microbiota plays a crucial role in health and has been linked to the development of colorectal cancer (CRC). Investigations have shown that SARS-CoV-2 infection causes changes to the gut microbiota, including an overall decline in microbial diversity, enrichment of opportunistic pathogens such as *Fusobacterium nucleatum* bacteremia, and depletion of beneficial commensals, such as the butyrate-producing bacteria. Further, these changes lead to increased colonic inflammation, which leads to gut barrier disruption, expression of genes governing CRC tumorigenesis, and tumor immunosuppression, thus further exacerbating CRC progression. Additionally, a long-lasting impact of SARS-CoV-2 on gut dysbiosis might result in a greater possibility of new CRC diagnosis or aggravating the condition in those already afflicted. Herein, we review the evidence relating to the current understanding of how infection with SARS-CoV-2 impacts the gut microbiota and the effects this will have on CRC carcinogenesis and progression.

## 1. Introduction

A novel coronavirus, severe acute respiratory syndrome coronavirus 2 (SARS-CoV-2), emerged late in 2019 in Wuhan, China, which led to a global pandemic resulting in over 170 million confirmed cases and over 3.5 million deaths attributed to this virus as of May 2021 [1]. SARS-CoV-2 is a positive-sense single-stranded RNA virus thought to be transmissible mainly by respiratory aerosol droplets and direct contact, which causes coronavirus disease 2019 (COVID-19) in humans [2]. The SARS-CoV-2 spike glycoprotein (S) protein S1 domain recognizes and binds the angiotensin-converting enzyme-2 (ACE2) receptor expressed on target cells, which facilitates attachment of the virus to the host cell [3]. Host protease TMPRSS2 subsequently cleaves S, revealing the S2 fusion domain, thus facilitating infection of the host cell [4]. It has been demonstrated that the ACE2 receptor and TMPRSS2 protease are required for viral binding to the cell and subsequent entry. However, other tissues have been shown to express differential receptors and proteases that can also interact with the spike protein, which could help explain the multi-tissue affinity of SARS-CoV-2 [5,6,7]. Upon infection, disease pathology involves multiple organs, including lung, gut, and brain. The symptoms range from mildly symptomatic with fatigue, headache, diarrhea, cough, and shortness of breath to acute respiratory distress syndrome and cytokine storm with multi-organ failure requiring mechanical ventilation. Immune dysregulation is a hallmark of COVID-19, paired with increased IL-6 and IL-13, which are potently prognostic [8]. The severity of the illness varies with co-existing comorbidities, biological sex, and age [9]. The potent inflammatory effects from this virus due to their immune system subverting non-structural proteins result in systemic pathologies in the severely ill. SARS-CoV-2 infections have unknown long-term consequences in patients with comorbidities, such as cancer [10].

Cancer patients are traditionally considered at high risk for respiratory viral infections due to their underlying immunosuppression [11]. Respiratory infections have a particularly lethal burden in cancer patients, given their reduced competency to fight infections due to altered immune responsiveness [11]. Infection with a respiratory syncytial virus (RSV), along with other family members of Coronaviridae, has been shown to inhibit and degrade p53, a tumor suppressor that can inhibit carcinogenesis [12,13]. This leads to the question, are cancer patients more likely to develop lethal complications after being infected by SARS-CoV-2? Early studies investigating the relationship between SARS-CoV-2 and cancer have shown higher mortality rates from SARS-CoV-2 infection in cancer patients than individuals without cancer [14]. While a massive effort to collect data on COVID-19 and cancer has been performed over the last year, the reported findings should be interpreted with caution. Specifically, patients with ongoing or recent treatment for hematologic, lung, or metastatic cancers are at higher risk of developing severe COVID-19 [14,15]. It is unclear how much of the increase in mortality rates is due to delayed diagnosis and suboptimal cancer management due to the pandemic [15].

SARS-CoV-2-caused immune dysregulation itself could provide even more significant complications for this already vulnerable subpopulation. Mechanistically, the interaction between the host immune environment and cancer or SARS-CoV-2 infections uses similar pathways, such as hypercoagulability, dysregulated immune response, elevated cytokine levels, altered expression ACE-2 and TMPRSS2, and prothrombotic status, which bring the human body into severe disequilibrium and may aggravate the effects of SARS-CoV-2 in some cancer patients [15]. Specific guidance regarding cancer treatment in COVID-19 patients is lacking due to minimal knowledge, particularly regarding differential immune responses.

SARS-CoV-2 infection begins with attachment to the host ACE2 receptor, with the primary site of infection being in the upper airway and lungs. However, the highest expression of ACE2 in the human body occurs in the intestinal enterocytes’ brush border [16]. A pan-cancer analysis found that ACE2 and TMPRSS2 are generally expressed less in tumors relative to normal tissue and that digestive organs (both tumor and normal samples) have the highest expression [17,18]. It has also been shown that colon epithelial cells express high levels of ACE2 and can support viral replication of SARS-CoV-2, resulting in gut barrier dysfunction [19]. Furthermore, the accessory host proteases required for spike processing and S2 domain exposure are present, including TMPRSS2 and TMPRSS4 [5]. The resulting local inflammation in the gut epithelia in conjunction with the systemic inflammation from the respiratory infection from SARS-CoV-2 can significantly impact the resident gut microbiota.

Influenza infection has been previously shown to significantly alter the composition of the intestinal microbiota [20]. Interferons produced in the lungs lead to the depletion of obligate anaerobic bacteria and proteobacteria enrichment in the gut, leading to dysbiosis. The action of these interferons was shown to inhibit the antimicrobial and inflammatory responses in the gut during Salmonella-induced colitis, which was shown to further enhance Salmonella intestinal colonization and dissemination, a risk factor for colon cancer [21]. Furthermore, patients with irritable bowel syndrome (IBD) had an increased risk for influenza, and IBD inflammation impacted the resolution of flu-like symptoms [22]. This is significant given that IBD is a prominent risk factor for CRC, and it is also known that the gut microbiota within this population is typically highly dysbiotic. It is known that dysbiosis of the gut can influence carcinogenesis and the progression of CRC [23,24]. A dysbiotic gut microbiota is incapable of properly signaling to distal tissues though bacterial metabolic by-products. This is further augmented alongside viral infections, which increase systemic inflammation, resulting in further dysbiosis to the symbiotic microbiome within the host [25]. In contrast to the dysbiosis leading to CRC, individuals with metastatic cancer were reported to regress, in response to viral infection, known as oncolytic therapy [26]. Additionally, another study reported transient remission of refractory NK/T-cell lymphoma during COVID-19 infection and the relapse after COVID-19 resolution in a single patient [27]. These reports together suggest SARS-CoV-2 immune responses have been shown to suppress NK cells cytotoxicity [28]. It has also been hypothesized that the antiviral CD8+ T cell immunity induced by SARS-CoV-2 infections could be used to enhance cancer immunotherapies [29]. While these studies provide the foundational premise for the impact of SARS-CoV-2, further understanding of how respiratory viral infections, particularly SARS-CoV-2, affect CRC progression represents a knowledge gap and critical unmet need.

Currently, little is known about the effect SARS-CoV-2 will have on the host microbiome, let alone on niche environments, such as the gut microbiota and its relation to CRC. According to the American Cancer Society, CRC is estimated to have caused over 140,000 new cases and 50,000 deaths in the United States in 2020 alone. A detailed understanding of how SARS-CoV-2 infection impacts the gut microbiota and CRC progression is of utmost importance [30].

## 2. The Gut Microbiome and CRC

CRC death remains one of the leading cancer-related mortalities in the United States, but the molecular mechanism of its development is not fully understood. Studies have found that in the United States, CRC incidence rates have increased by 2% annually among adults younger than age 55 from 2007 to 2016 [30] that has led to the recommendation to lower the age for starting colonoscopic screening for CRC. In the past decade, the Human Microbiome Project’s initiative by the National Institutes of Health has highlighted the importance of the gut microbiota to human health. The gut microbiota plays a crucial role in health through its protective, trophic, and metabolic activities. The gut microbiota has been recently linked to CRC development, as microbiota-derived metabolites have been shown to influence carcinogenesis directly [31].

The anaerobic microbes ferment undigested dietary components that reach the large intestine to produce a wide range of metabolites, with the significant fermentation products in healthy adults being gases and organic acids: short-chain fatty acids (SCFAs), such as acetic acid, propionic acid, and butyric acid and medium-chain fatty acids (MCFAs), such as linoleic acid, lauric acid, and oleic acid [32]. They are then released into the colonic lumen and function as signaling molecules between the bacteria and the host [31]. SCFAs have also been shown to maintain the gut barrier and produce an anti-inflammatory effect by introducing Treg cell differentiation and the expression of anti-inflammatory cytokines [33]. Importantly, these activities in the gut form a multi-organ axes, such as the “gut–lung axis” and the “gut–brain axis”, which are dependent on the metabolic products of the gut in maintaining the healthy homeostatic status of these organ systems [34,35].

Gut dysbiosis allows bacterial metabolomes and products to enter the circulatory system, leading to systemic inflammation [36]. The fecal analysis identifies marked dysbiosis in CRC patients from healthy cohorts [23]. Dysbiosis of the gut microbiota can influence immune status, carcinogenesis, and tumor progression in CRC [37,38]. CRC studies have demonstrated continual alterations in the gut microbiota during tumor development, and these alterations are directly responsible for tumor progression [39]. In one study, the colonization of germ-free mice with microbiota from tumor-bearing mice significantly increased tumorigenesis [40].

**Figure 1:**
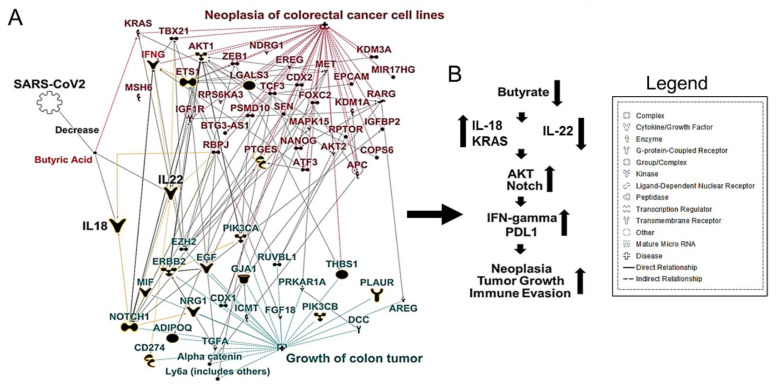
SARS-CoV-2 Infection: Implications for CRC. (**A**) Ingenuity Pathway Analysis (IPA)-generated network map showing known connections among butyrate, IL-18, IL-22, and CRC in IPA. (**B**) Simplified schematic of (**A**), showing gut dysbiosis caused by a decrease in butyrate-producing bacteria and a subsequent lack of IL-22 production, which allows bacterial metabolites to enter the circulatory system leading to an increase in inflammatory markers, thus increasing colitis-associated colon cancer.

Typical findings in CRC patients are a marked decrease in *Firmicutes*, *Bacteroides*, and *Actinobacteria*, paired with increases in *Fusobacterium* and *Porphyromonas* populations [41]. *Actinobacteria* are one of the four major phyla of the gut microbiota. Although they represent only a small percentage of the bacterial community, *Actinobacteria* are pivotal in maintaining gut homeostasis and immune tolerance [42]. Unbalanced populations of *Actinobacteria* have been evidenced in several pathological conditions [43]. Specifically for CRC, the reduction in metabolites associated with the *Actinobacteria* phylum paired with a proinflammatory, cellular-mediated, cytotoxic T-helper cell-1 (Th1) immune response induced by this group lends itself to a poor prognosis of CRC [44]. A decrease in butyrate-producing bacteria (especially the *Actinobacteria* and *Firmicutes* phyla) also decreases the primary energy source for colonocytes. It increases intra-colonic pH, creating a hostile environment for colonocytes and contributing to tumorigenesis [41]. To further explore the relationship between butyrate and CRC, we used Ingenuity Pathway Analysis (IPA) [45] that uses a database of prior biological knowledge derived from analyzing and annotating the peer-reviewed scientific literature. We used the My Pathway/Path Designer tools in IPA to plot known interactions between butyrate and CRC. We found that gut dysbiosis could cause a decrease in butyrate-producing bacteria. A subsequent lack of IL-22 production allows bacterial metabolites to enter the circulatory system, leading to increased inflammatory cell infiltration, thus increasing colitis associated with colon cancer (Figure 1A,B).

Consumption of a high-fat and -protein diet has been linked to increased secretion of bile acids. Certain microbes convert into primary and secondary bile acids, which are toxic and promote tumorigenesis [46]. Bile acids are key signaling molecules that regulate digestive functions and physiological functions such as glucose and lipid metabolism and immune homeostasis [47]. Bile acids are synthesized from cholesterol and conjugated to glycine or taurine in hepatocytes. Most bile acids are secreted into the small intestine. However, bile acids that reach the large intestine will interact with gut microbes. Bile acids are toxic for many gut bacteria. Thus, high levels will put selective growth pressure on our gut microbiota, favoring certain bacteria in our gut to act enzymatically on the bile acids [47]. For example, excess taurine is excreted as a conjugated bile acid called taurocholic acid and converted into deoxycholic acid, which studies have shown to be genotoxic and promote tumor formation [48]. In studies, deoxycholic acid can activate cellular signaling pathways associated with cell proliferation and apoptosis [46]. A major enzyme in the production of deoxycholic acid, 7α-dehydroxylation, has been characterized in species belonging to the genera *Eubacterium* and *Clostridium*, including the species *Clostridium scindens* and *Clostridium hylemonae* [47].

It has been shown in mice that increasing the proportion of *Lactobacillus* and *Bifidobacterium* in the gut microbiota through probiotic supplementation (oral gavage daily of 0.6 billion CFU (colony forming units) each of *Lactobacillus acidophilus*, *Lactobacillus rhamnosus,* and *Bifidobacterium bifidum*, diluted in 200 μL of drinking water) reduced inflammatory cell infiltration, lowered chemokine expression, and reduced colitis-associated CRC [49]. Other studies have suggested that an expansion of *Proteobacteria*, usually a minor constituent of the gut microbiota, is a potential microbial signature of epithelial dysfunction observed in patients with CRC [39,50,51]. A complete understanding of the complex factors that lead to dysbiosis has not yet materialized, and the effect of this on CRC remains unclear.

## 3. SARS-CoV-2 Induced Gut Microbiome Dysbiosis and CRC

Although the most common COVID-19 symptoms are respiratory, SARS-CoV-2 infections also target the gastrointestinal tract, culminating in inflammation and intestinal cramps to diarrhea [52]. One hypothesis is that ACE2 downregulation, caused by SARS-CoV-2 infection, decreases activation of the mechanistic target of rapamycin (mTOR) and increases autophagy, leading to intestinal dysbiosis and diarrhea [52]. ACE2 expression in lungs is downregulated in wild-type mice infected with SARS-CoV and mice injected with recombinant SARS spike protein. This downregulation may play a role in SARS pathogenesis and disease progression to ARDS [53,54,55]. Another hypothesis is that the small bowel is likely a key site of amplification of the systemic inflammatory response, where blockage of ACE2 causes increased levels of angiotensinogen and hyperactivation of the renin–angiotensin system, leading to a shutdown of the amino acid transporter BA0T1 and a subsequent lack of cellular tryptophan, which leads to decreased secretion of antimicrobial peptides and gut dysbiosis [56].

**Figure 2:**
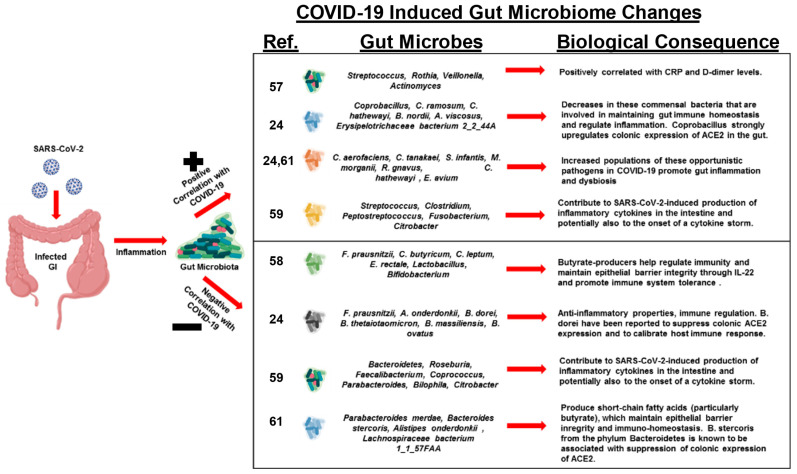
Schematic showing changes in the gut microbiome caused by SARS-CoV-2 infection, their correlation with COVID-19 severity, and the biological consequences of these changes [24,57,58,59,61].

Recent studies have observed that patients with COVID-19 develop a dysbiotic microbiota of the gut [24,57,58,59,60,61] (Figure 2). One study investigated changes in fecal microbiomes of patients with SARS-CoV-2 infection during hospitalization and associations with severity and fecal shedding of the virus [24]. Patients with COVID-19 had significant alterations in their fecal microbiomes, characterized by enrichment of opportunistic pathogens and depletion of beneficial commensals at the time of hospitalization and at all time points during hospitalization. Depleted symbionts and gut dysbiosis persisted throughout hospitalization, even after clearance of SARS-CoV-2 and resolution of respiratory symptoms. Throughout hospitalization, *Bacteroides dorei*, *Bacteroides taiotaomicron*, *Bacteroides massiliensis*, and *Bacteroides ovatus*, which downregulate the expression of ACE2 in the murine gut, correlated inversely with SARS-CoV-2 load in fecal samples from patients. Interestingly, the baseline abundance of specific microbes in the gut, such as *Coprobacillus, Clostridium ramosum,* and *Clostridium hathewayi*, correlated with COVID-19 severity. There was an inverse correlation between the abundance of *Faecalibacterium prausnitzii*, an anti-inflammatory bacterium, and disease severity.

Another study investigated the changes in abundance of ten predominant intestinal bacterial groups in COVID-19 patients to establish a correlation between these bacterial groups and clinical indicators of pneumonia [58]. The results indicate that dysbiosis occurred in COVID-19 patients, and changes in the gut microbial community were associated with disease severity and hematological parameters. The number of common opportunistic pathogens, *Enterococcus* (Ec) and *Enterobacteriaceae* (E), were shown to be increased in COVID-19 patients, especially in the critically ill. Observations suggest that these bacterial groups can serve as diagnostic biomarkers for COVID-19 and that the Ec/E ratio can be used to predict death in critically ill patients.

The next study investigated the transcriptional activity of SARS-CoV-2 and its association with fecal microbiome alterations in COVID-19 patients [61]. Even in the absence of gastrointestinal (GI) symptoms, some patients continued to display an active viral infection signature up to 6 days after clearance of SARS-CoV-2 from respiratory samples. Fecal samples with a high SARS-CoV-2 signature had higher abundances of the bacterial species *Collinsella aerofaciens*, *Collinsella tanakaei*, *Streptococcus infantis*, and *Morganella morganii*. The gut microbiota of patients with active SARS-CoV-2 GI infection was characterized by enrichment of opportunistic pathogens, loss of salutary bacteria, increased functional capacity for nucleotide and amino acid biosynthesis, and carbohydrate metabolism. This study provides evidence for a dormant, prolonged GI infection by SARS-CoV-2, even in the absence of GI symptoms and after recovery from respiratory infection of SARS-CoV-2.

COVID-19 patients have also been shown to have significantly reduced bacterial diversity, a higher relative abundance of opportunistic pathogens, such as *Streptococcus, Rothia*, *Veillonella*, and *Actinomyces*, and a lower relative abundance of beneficial symbionts [57,59]. Levels of *Fusicatenibacter*, *Romboutsia*, *Intestinibacter*, *Actinomyces*, and *Erysipelatoclostridium* showed high accuracy for distinguishing COVID-19 patients, which suggests the potential value of the gut microbiota as a diagnostic biomarker and therapeutic target for COVID-19. Reduced bacterial diversity in the gut has been associated with acute and long-term metabolic effects and disease propensities.

**Figure 3:**
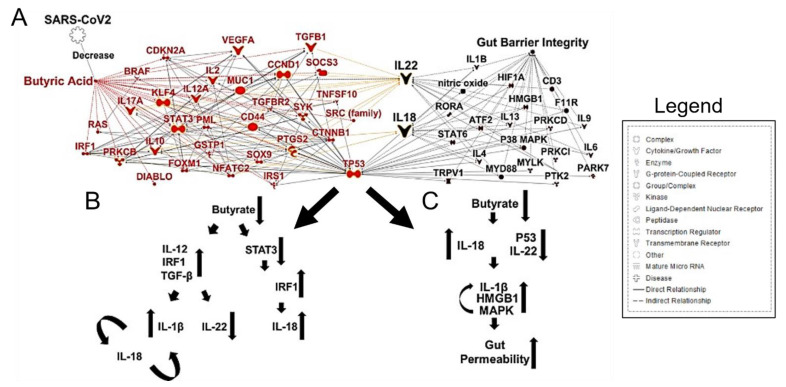
SARS-CoV-2 Infection, Butyrate, and Gut Barrier Integrity. (**A**) Ingenuity Pathway Analysis (IPA) (Qiagen)-generated network map showing known connections among butyrate, IL-18, IL-22, and gut barrier integrity. (**B**,**C**) A reduction in butyrate-producing bacteria disrupts gut epithelial barrier integrity via IL-22 signaling and promotes inflammatory signaling through IL-18.

In patients with COVID-19, the abundance of butyrate-producing bacteria, such as *Faecalibacterium prausnitzii*, *Clostridium butyricum*, *Clostridium leptum*, and *Eubacterium rectale*, decreased significantly, and this shift in the bacterial community may help discriminate critically ill patients from patients that have a more moderate disease state [58]. Fecal samples from patients with low-to-no SARS-CoV-2 infectivity had higher abundances of SCFA-producing bacteria, *Parabacteroides merdae, Bacteroides stercoris, Alistipes onderdonkii,* and *Lachnospiraceae bacterium 1_1_57FAA* [61]. Importantly, butyrate-producing bacteria are of critical importance in maintaining gut barrier integrality. SCFAs have been implicated in signaling for IL-22, which maintains gut and lung epithelial barrier integrity [62]. Butyrate and other SCFAs regulate inflammation by macrophages in the intestine and promote the Warburg effect, which metabolically constrains the neoplastic cells [63]. CRC has a metabolic dependence on anaerobic glycolysis; thus, fatty acid oxidation is limited. Given this metabolic state, butyrate begins to accumulate in the cytoplasm of cancerous colonocytes, allowing this SCFA to act as an HDAC inhibitor, sensitizing the cancerous colonocytes to apoptotic pathways [64,65]. Specifically, a reduction in butyrate-producing bacteria and gut dysbiosis contributed to less IL-22 production, which is integral to maintain both gut and lung epithelial barrier integrity [66] (Figure 3A,B).

**Figure 4:**
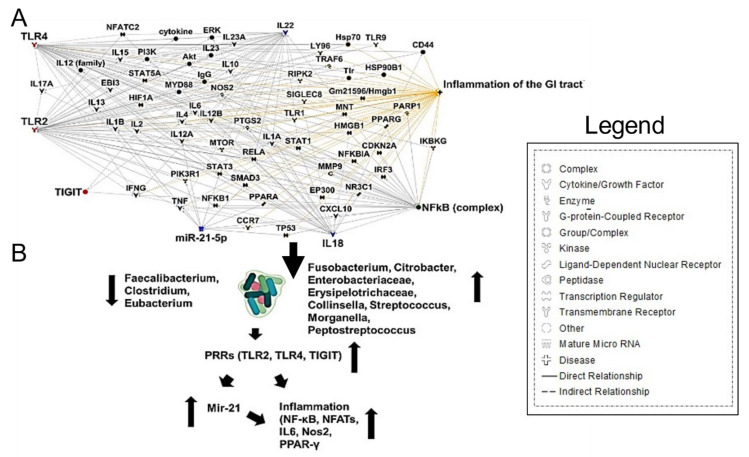
*Fusobacterium nucleatum* Bacteremia Causes Gut inflammation. (**A**) Ingenuity Pathway Analysis (IPA)-generated network map showing pathway responsible for the *F. nucleatum* induction of inflammation. (**B**) *F. nucleatum* interacts with Toll-like receptors 2 and 4, resulting in increased miRNA-21 expression, which promotes NF-kB induction and contributes to the induction of inflammation.

*Fusobacterium nucleatum* bacteremia has also been observed in severe COVID-19 cases and shown to colonize colon mucus with associated mucosal inflammation [67]. Along with the immune dysfunction seen in COVID-19, *F. nucleatum* causes immunosuppression by the action of one of its surface proteins, Fap2, which has been recognized as a galactose-binding lectin that can interact with the T cell ITIM domain (TIGIT) inhibitory receptor [68]. Furthermore, *F. nucleatum* was shown to interact with Toll-like receptors 2 and 4 signaling, resulting in increased microRNA-21 (miRNA21) expression, which promotes NF-kB induction, contributing to inflammation and cell proliferation [68] (Figure 4A,B). The intestinal inflammatory cytokine IL-18 was also shown to be elevated in the serum of COVID-19 patients [59]. These results indicate that gut microbiota composition changes contribute to the SARS-CoV-2-induced inflammatory cytokines production in the intestine.

Interestingly, one study also investigated changes in the fecal fungal microbiomes (mycobiome) of COVID-19 patients [69]. Patients with COVID-19 had significant alterations in their fecal mycobiomes, characterized by increased proportions of opportunistic fungal pathogens, *Candida albicans*, *Candida auris*, and *Aspergillus flavus*. Two respiratory-associated fungal pathogens, *A. flavus* and *Aspergillus niger*, were detected in fecal samples from a subset of patients with COVID-19, even after clearance of SARS-CoV-2 from nasopharyngeal samples and resolution of respiratory symptoms. These results are striking and re-enforce the commensal nature of all microbes, which may encompass bacteria, fungi, protists, and viruses alike.

There are increasing reports of persistent and prolonged effects after COVID-19, now termed “long haulers”, a syndrome characterized by persistent symptoms and delayed or long-term complications beyond 4–6 weeks from the onset of symptoms [70,71]. This may be caused by the observed post-acute COVID-19 syndrome seen in some cases, where immune dysregulation and gastrointestinal symptoms are still ongoing even after clearance of SARS-CoV-2, thus impacting the status of the patient’s microbiome [72,73]. Due to the recent nature of the COVID-19 pandemic, the time of restoration of normal gut microbiota has yet to be thoroughly investigated, so the timeline and long-term effects of dysbiosis are unknown and require further studies.

A well-known respiratory infection phenomenon is gut-barrier dysfunction, which is correlated with a more severe clinical course of the disease [74]. Clinically, it has been shown that modulating the gut microbiota can slow early influenza virus replication in lung epithelia, resulting in reduced enteritis and ventilator-associated pneumonia [74]. Expansion of *Bifidobacterium* species upon and after influenza infection, for example, has been shown to be protective from the inflammation that could cause dysbiotic events [75,76]. Currently, there is no direct clinical evidence that the modulation of the gut microbiota plays a therapeutic role in the treatment of COVID-19. However, clinical trials of probiotics against COVID-19 are underway [77]. Probiotics can help patients maintain immune homeostasis in the gut and avoid overactivation of the immune response by decreasing proinflammatory signaling and maintaining gut barrier integrity.

The human immune system has evolved to cope with the presence of microorganisms both inside our body and out [78]. This fact means that most infectious diseases caused by viruses or bacteria are self-limiting [79,80]. This could be one possible reason for the asymptomatic or mild nature of most COVID-19 cases [81]. These microorganisms can serve as a source of metabolites, such as essential amino acids and fatty acids, for the human body [82]. In a state of gut dysbiosis, a transient nutritional supply excess may occur, as both the microorganisms and the damaged host tissue will be degraded and become a possible source of nutrition. This may promote hyper inflammation during acute infection [83] and possibly lead to chronic diseases, such as cancer [84]. For example, some of the excess nutrition from damaged tissue, together with the excess nutrition from a dysbiotic gut microbiome, could be turned into lipid intermediates, causing lipotoxicity and further tissue damage [85]. This highlights how the nutritional state can impact a person’s health and disease outcome. As such, food intake restriction could offer a promising way to control chronic inflammation [86].

**Figure 5:**
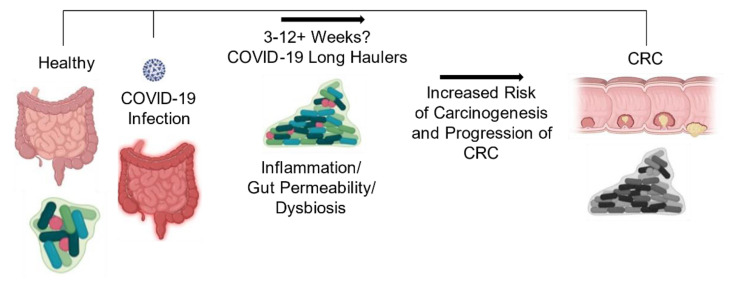
A schematic showing COVID-19-Induced Gut Microbiome Changes Increase the Risk of Carcinogenesis and Progression of CRC. COVID-19 infection leads to an increased risk of CRC carcinogenesis and progression.

Together, these collective observations warrant greater investigation into the relationship between changes in the gut microbiota that encompass all microorganisms due to COVID-19 infection and associations with increased risk for CRC development or progression (Figure 5). Given the widespread incidence of this virus and that CRC is the third most common cancer diagnosed in the United States in both men and women, studies investigating the associated effects of these two diseases are in great need. Upon elucidating answers to these questions, it may be possible to develop effective probiotic therapies that maintain a more homeostatic gut environment during the disease, which could help improve CRC survival rates.

## 4. Conclusions and Future Directions

**Figure 6:**
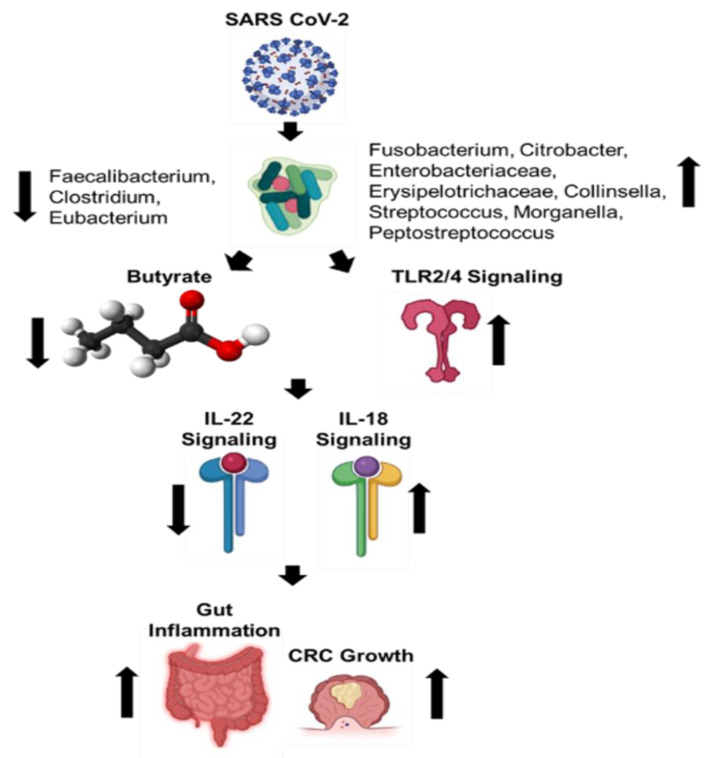
A summary COVID-19-Induced Gut Microbiome Changes Increase the Risk of Carcinogenesis and Progression of CRC. Changes to the gut microbiota caused by SARS-CoV-2 lead to increased expression of genes involved in the inflammatory response, resulting in gut barrier disruption and increased risk of CRC.

The SARS-CoV-2 pandemic has left many unanswered questions on the long-term impacts of this virus post-infection. This question is of particular importance to those with underlying comorbidities before the onset of COVID-19. Cancer is just one of many of said comorbidities. Still, the multifaceted nature of this disease and immunosuppressive treatments make understanding the role of COVID-19 in relation to cancer to be of the utmost importance. Once age is considered, those born in 1990 have about double the risk of colon cancer than people born around 1950 [30]. Factors such as dietary changes, greater preservatives in foods, and changes in hygiene practices may contribute to this observable phenomenon. The gut microbiota plays a crucial role in health and has been linked to CRC development [31]. Early investigations have shown that the gut microbiota and mycobiota are significantly altered in patients with COVID-19. Changes to the gut microbiota caused by SARS-CoV-2 infection include enrichment of opportunistic pathogens and depletion of beneficial commensals, an overall decline in microbial diversity, a loss of butyrate-producing bacteria, and *F. nucleatum* bacteremia. In both CRC and SARS-CoV-2 infection, the increased expression of genes involved in the inflammatory response leads to a worsening in gut barrier dysfunction. Changes caused by SARS-CoV-2 infection may further exacerbate CRC progression through increased expression of CRC tumorigenesis markers, tumor immunosuppression, and the induction of inflammation, leading to gut barrier disruption and worsening CRC progression (Figure 6).

A complete understanding of the complex factors leading to dysbiosis has not yet materialized. It is critically important that we understand how infection with SARS-CoV-2 will impact the gut microbiota and CRC progression. The impact of SARS-CoV-2 on gut dysbiosis and the gut microbiota may be long-lasting, thus underscoring the importance of restoring a competent microbiome to resist the development of GI diseases, such as irritable bowel syndrome, allergic colitis, recurrent *Clostridioides difficile* infection, and CRC [87,88].
